# Physician Assistant Distribution in Texas-Mexico Border Counties: Public Health Implications

**DOI:** 10.1155/2010/975016

**Published:** 2010-12-28

**Authors:** P. Eugene Jones, Karen E. Mulitalo

**Affiliations:** ^1^Department of Physician Assistant Studies, University of Texas Southwestern Medical Center at Dallas, 5323 Harry Hines Boulevard, Suite V4.114, Dallas, TX 75390-9090, USA; ^2^The University of Queensland School of Medicine Mayne Medical School, Building 288 Herston Road, Herston, QLD 4006, Australia

## Abstract

*Purpose*. Texas Medical Board physician assistant (PA) data were assessed to assist workforce education and planning strategies for PA programs in regions with high percentages of Hispanic populations. *Methods*. Data were assessed for gender, ethnicity, program attended and current employment addresses within the 14 Texas-Mexico border counties. *Results*. Of the 329 border county PAs, 227 self-reported as Hispanic (69%), and 53% were female. Remarkably, 72% of all Hispanic PAs attended two of the six public Texas PA Programs. *Conclusions*. The Sullivan Commission report of 2004 concluded that the primary cause of poor public health care for minorities resulted from unequal representation of minorities in the health care professions. Two public Texas PA programs have made substantial contributions to public health care access in poverty-stricken border areas by educating and placing Hispanic PAs within medically underserved communities.

## 1. Introduction

Between the years 1990 and 2010, the US Hispanic population has doubled, accounting for 39% of the total US population growth [1]. In addition to the changing Hispanic demographics, the overall rapid increase of international immigrants over the past several years has led to additional government initiatives to achieve *Healthy People 2010 *health care access objectives [2]. The national goals of increasing access to primary health care services and reducing health care disparities are affected by the health education community's ability to train providers whose cultural and language diversity help address a demographically changing patient population. In *A Strategic Plan for the Physician Assistant Profession*, the factors predicted to influence success for the PA profession in the 21st century mirror characteristics identified as key to the success of *Healthy People 2010* objectives, including the ability to facilitate a transfer of knowledge to patients in a culturally appropriate manner. 

It has been determined that one of the more important sources of health information for people of Hispanic heritage is the physician or nurse. The inability of these health care providers to speak Spanish is cited by Hispanics as the number one barrier to health care [3–5]. Doctor-patient language concordance has also been shown to make Hispanics more likely to keep office appointments, adhere to medication regimens, ask more questions, recall recommendations, and show improved overall health status [6]. 

The numbers of Hispanics, Latinos, and others who primarily speak Spanish grow daily. However, access to health care services for this group may be restricted due to language and cultural barriers. In Texas, 27% of the population over the age of five speaks Spanish at home [7]. With the close proximity to Mexico and large number of Spanish-speaking immigrants, Texas is one of the areas most deprived of primary health care access in the southwest. Here some migrant workers and immigrants live in *colonias* (makeshift shacks usually without running water and often amidst unsanitary conditions) and experience higher than usual illness rates, greater health care needs, and increasing costs to society [8]. 

Research in the areas of language and cultural competency has shown that health literacy is limited when health care providers are neither fluent in the patient's language nor aware of the cultural implications [9]. Perception of care and compliance with instructions may be related to how well the provider communicates with patients [10]. The delivery of health care services is enhanced when language and cultural differences are overcome through translation, interpretation or, ideally, when the health care provider speaks the language of the patient [10]. In some instances, especially when dealing with very personal health matters, an interpreter may inaccurately translate the clinician's intention, eroding the credibility of the provider [11]. When health care services are not readily attainable because of communication gaps such as this between clinician and patient, the health of the individual suffers either through health illiteracy or preventable illness [12]. An important strategy to improve health care delivery in medically underserved US-Mexico border communities has been the focused placement of Hispanic health care professionals in these areas. The purpose of this study was to examine the training program origin and distribution of Hispanic PAs for clinical practice within Texas-Mexico border counties. Public health care access and policy development requires examination and dissemination of targeted educational outcomes data. 

## 2. Materials and Methods

**Figure 1:**
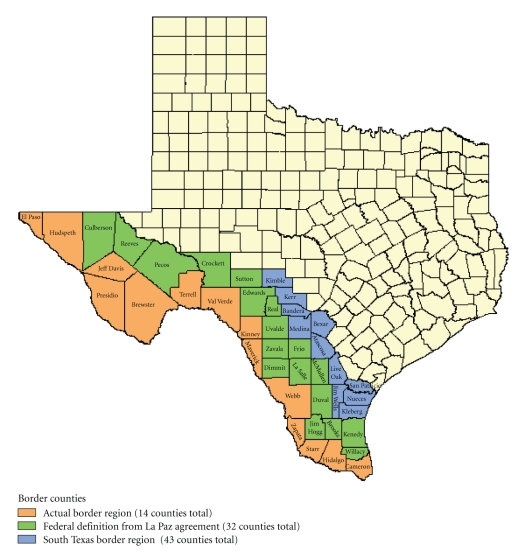
Texas-Mexico border region.

Although the physical border between Texas and Mexico comprises 14 Texas counties (Figure 1), in order to address broader public health and environmental issues the Federal definition was extended to include an additional 18 counties in the 1983 La Paz Agreement on Cooperation for the Protection and Improvement of the Environment in the Border Area [13]. For purposes of identifying PA practice along the actual physical border, we restricted the study to the actual 14 international border counties. A review of Texas Medical Board PA practice demographics along the Texas-Mexico border was then conducted to provide insight for future curriculum development and admissions strategies for PA programs located in a state with a high percentage Hispanic population. The Texas Medical Board database includes 27 descriptive items per licensed PA. The data were reviewed in May 2010 to identify self-reported gender, ethnicity, PA program attended and current employment addresses within the 14 Texas-Mexico border counties. The data were compared to a previous study that used US Census Bureau data to examine PA workplace data for these counties [14]. The data were also compared with 2008 State of Texas Population Estimates for the same 14 Texas counties as well as the US Census 2000 data. 

## 3. Results

The 2010 average Hispanic population in the 14 Texas-Mexico border counties was 87.3%, compared with the US average of 15.1% [15]. By comparison, according to 2009 American Academy of Physician Assistants census data, Hispanics represented only 3.6% of the US PA total [16]. According to the US Census 2000 data study, there were 224 PAs in the 14 border counties, and that number has since increased 32% to 329. Of the 329 border county PAs, 227 self-reported as Hispanic (69%), and 53% were female. Distribution of Hispanic PAs among the border counties reflected the county population distribution as a whole, ranging from zero Hispanic PAs in half the counties, to 128 Hispanic PAs in Hidalgo County, the most heavily populated border county. In Maverick and Starr Counties, 100% of all employed PAs were Hispanic (Table 1). Remarkably, 72% of all Texas-Mexico border county Hispanic PAs attended one of two public Texas PA Programs, either University of Texas-Pan American (52%) or University of Texas Medical Branch-Galveston (20%).

## 4. Conclusions

The Sullivan Commission report of 2004 concluded that the primary cause of poor public health care for minorities resulted from unequal representation of minorities in the health care professions. This analysis of PA practice demographics within Texas-Mexico border counties reveals a greater percentage of Hispanic PAs when compared with other ethnicities and with statewide and national averages. The findings also show that two public Texas PA programs have made substantial contributions to health care access in poverty-stricken border areas by educating and placing Hispanic PAs within medically underserved communities. As the Hispanic population continues to increase, these data support the continuing need to develop curricula that increases cultural competency and proficiency in the Spanish language among PA graduates as well as suggesting that the practice patterns of underrepresented minority PA graduates can benefit underserved populations.

## Figures and Tables

**Table 1:** 

County	Total physician assistants	Hispanic physician assistants	Percent Hispanic physician assistants
Brewster	2	0	0
Cameron	53	38	72
El Paso	86	41	48
Hidalgo	161	128	80
Hudspeth	0	0	0
Jeff Davis	0	0	0
Kinney	0	0	0
Maverick	6	6	100
Presidio	1	0	0
Starr	8	8	100
Terrell	0	0	0
Val Verde	5	1	20
Webb	6	5	83
Zapata	1	0	0

Total	329	227	69% (mean)
